# Development of Folate-Functionalized PEGylated Zein Nanoparticles for Ligand-Directed Delivery of Paclitaxel

**DOI:** 10.3390/pharmaceutics11110562

**Published:** 2019-10-30

**Authors:** Zar Chi Soe, Wenquan Ou, Milan Gautam, Kishwor Poudel, Bo Kyun Kim, Le Minh Pham, Cao Dai Phung, Jee-Heon Jeong, Sung Giu Jin, Han-Gon Choi, Sae Kwang Ku, Chul Soon Yong, Jong Oh Kim

**Affiliations:** 1 College of Pharmacy, Yeungnam University, 214-1, Dae-Dong, Gyeongsan 712-749, Korea; zarchisoeygn96@gmail.com (Z.C.S.); owqcn@foxmail.com (W.O.); gtmmilan2@gmail.com (M.G.); poudel.kishwor99@gmail.com (K.P.); kyunsbk14@naver.com (B.K.K.); phamleminh87@gmail.com (L.M.P.); phungcaodai@gmail.com (C.D.P.); jeeheon@yu.ac.kr (J.-H.J.); 2 Department of Pharmaceutics, University of Pharmacy (Mandalay), Mandalay-Lashio Rd, Mandalay 05011, Myanmar; 3 Department of Pharmaceutical Engineering, Dankook University, 119 Dandae-ro, Dongnamgu, Cheonan 31116, Korea; sklover777@gmail.com; 4 College of Pharmacy, Institute of Pharmaceutical Science and Technology, Hanyang University, 55 Hanyangdaehak-ro, Sangnok-gu, Ansan 426-791, Korea; hangon@hanyang.ac.kr; 5 Department of Anatomy and Histology, College of Korean Medicine, Haany University, Gyeongsan 712-715, Korea

**Keywords:** folic acid, paclitaxel, nanoparticle, zein

## Abstract

In this study, we investigated the active targeted delivery of a hydrophobic drug, paclitaxel (PTX), via receptor-mediated endocytosis by folate receptors expressed on cancer cells using a protein-based nanoparticle system. PTX was loaded on zein nanoparticles and conjugated with folate (PTX/Zein-FA) to estimate its chemotherapeutic efficacy in folate receptor-expressing KB cancer cells. PTX/Zein-FA nanoparticles were successfully developed, with a nanoparticle size of ~180 nm and narrow polydispersity index (~0.22). Accelerated release of PTX in an acidic environment was observed for PTX/Zein-FA. An in vitro cellular study of PTX/Zein-FAs in KB cells suggested that PTX/Zein-FA improved the cytotoxic activity of PTX on folate receptors overexpressed in cancer cells by inducing proapoptotic proteins and inhibiting anti-apoptotic proteins. In addition, PTX/Zein-FA exhibited anti-migratory properties and could alter the cell cycle profile of KB cells. A549 cells, which are folate receptor-negative cancer cells, showed no significant enhancement in the in vitro cellular activities of PTX/Zein-FA. We describe the antitumor efficacy of PTX/Zein-FA in KB tumor-bearing mice with minimum toxicity in healthy organs, and the results were confirmed in comparison with free drug and non-targeted nanoparticles.

## 1. Introduction

Active ligand-mediated tumor targeting has emerged as one of the most beneficial tools for safe and effective delivery of chemotherapeutic agents with maximum therapeutic efficacy and negligible systemic side effects during chemotherapy [1,2]. An improved efficacy of targeted drug delivery systems can be achieved by using nanoparticles that have been functionalized via surface modification [3]. Several targeting moieties can be attached on nanoparticle carriers’ surface to transfer the chemotherapeutic agents to the targeted tumor area via receptor-mediated endocytosis [4]. Specific ligands, such as antibodies, aptamers, peptides, and small vitamin molecules, upon conjugation with nanoparticles, can support drug delivery to optimize chemotherapy treatment [5]. Folic acid (FA) has been widely utilized as a target molecule in targeted drug delivery systems [6]. FA is superior to the other moieties because it is a naturally occurring compound with a low molecular weight and shows ease of synthesis, higher immunogenicity [7], and easy association with PEG, without disturbing the binding activity of receptors and drug carriers [8,9].

Paclitaxel (PTX) is widely used as a chemotherapeutic agent against many kinds of cancers [10]. PTX can inhibit mitotic pathways, as it stabilizes microtubules and causes reduction in the dynamic pattern of cytoskeletal structures [11]. It promotes tubulin dimerization and inhibits microtubule depolymerization, thereby increasing antitumor efficacy [12]. PTX is observed to be superior than other antimicrotubular agents, such as vinblastine, in the treatment of bladder cancer [13]. Although its therapeutic efficacy has been proven, clinical application of PTX is limited due to its low solubility in water; furthermore, it causes hypersensitivity, nephrotoxicity, and neurotoxicity [14]. Several nano-sized drug carriers, e.g., liposomes, micelles, and polymeric nanoparticles have been suggested to safely deliver and minimize the untoward effects of PTX [15].

Among the different types of nanoparticles, protein-based nanoparticles show a higher impact in chemotherapy as drug carriers, because of their smaller size distribution and ease of preparation [16]. Furthermore, these can also be smoothly modified for the incorporation of targeting moieties on its surface [17] and display inherent properties, such as biocompatibility, biodegradability, digestion in the body, and absorption of cargo materials via electrostatic interactions [18]. Of late, a natural protein polymer, zein, derived from corn, has received great attention for the development of a drug delivery nanocarrier system and has been accepted as a safe and attractive biomaterial by the FDA. [19]. Zein can be used as a controlled release drug carrier, as it has already been proven by many researchers as a biodegradable agent with a delayed rate of degradation [20]. Hydrophobic molecules, polypeptides, and amino acids, such as proline and glutamine, which are constituents of zein, improve the loading capacity of hydrophobic anticancer drugs [21].

**Figure 1:**
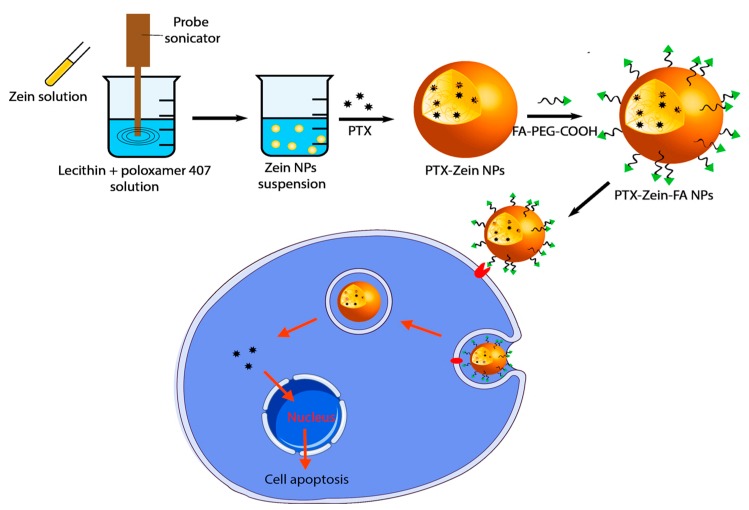
Schematic illustration of the preparation of folate-conjugated zein nanoparticles to deliver paclitaxel to folate receptor-expressing KB cells.

In this study, we used an amphiphilic zein nanoparticle system to create the core for PTX, and folate-targeted DSPE-PEG was used to deliver the maximum amount of drug PTX to a targeted cancer area, i.e., the tumor area, via receptor-mediated endocytosis (Figure 1), with minimum toxicity to healthy organs.

## 2. Materials and Methods

### 2.1. Materials

PTX was purchased from LC Laboratories (Boston, MA, USA). Zein, amine-PEG-carboxylic acid, folic acid (FA), 3-(4,5-dimethylthiazol-2-yl)-2,5-diphenyltetrazolium bromide (MTT), 1-ethyl-3-(3-dimethyl amino propyl) carbodiimide hydrochloride (EDC), N-hydroxy succinimide (NHS), triethylamine (TEA), and dimethyl sulfoxide (DMSO) were purchased from Sigma-Aldrich (St. Louis, MO, USA). KB and A549 cells were obtained from the Korean Cell Line Bank (Seoul, Korea). Coumarin-6 was purchased from Thermo Fisher Scientific Inc. (Waltham, MA, USA). Primary antibodies against Bax, Bcl2, caspase 3 and 8, p27, and p53 were obtained from Cell Signaling Technology Inc. (Danvers, MA, USA). All the other reagents were of reagent grade and used without further purification.

### 2.2. Synthesis and Characterization of Folate-PEG

For the synthesis of folate-PEG conjugated nanoparticles, firstly, the carboxylate group of FA was activated by NHS and N’,N’-dicyclohexyl carbodiimide (DCC) for conjugation with the amine group of bifunctional PEG (Figure S1A) [22]. Briefly, a mixture of FA (1.5 g) and DMSO (20 mL) was reacted with DCC (0.65 g) and NHS (1 g) for 12 h at 25 °C in a nitrogenous atmosphere. A solution of NH_2_–PEG–COOH (0.65 g) and DMSO (5 mL) was mixed with activated FA and kept for 5 h at 25 °C in a nitrogenous atmosphere. The resultant solution was diluted with acetone (45 mL) (non-solvent for FA) and centrifuged; subsequently, the supernatant was dialyzed using a dialysis bag (Spectra Por 6, MW cutoff, 1000) in deionized water; finally, it was freeze-dried and stored until use in the preparation of PTX/Zein-FA. Before it had been used in the experiments, the yellowish freeze-dried powder of FA–PEG–COOH was characterized for chemical purity by Fourier-transform infrared spectroscopy (FTIR), X-ray diffraction (XRD), ^1^H NMR, and proton nuclear magnetic resonance (Varian Inc., Palo Alto, CA, USA) analysis, using d_6_-DMSO as a solvent (Figure S1B). The amount of FA conjugated in FA–PEG–COOH was calculated by using the standard curve of free FA, which was measured at 365 nm using an ultraviolet (UV) spectrophotometer (PerkinElmer U-2800, Hitachi, Tokyo, Japan) [23]. The absorbance of the polymer-conjugated FA was also measured under the same UV-visible spectroscopy conditions for free FA (Figure S1C).

### 2.3. Preparation and Characterization of PTX/Zein-FA

PTX/Zein-FA was prepared using solvent evaporation method [24]. The solution of zein (30 mg) and ethanol (15 mL) was added to a surfactant composed of lecithin and poloxamer 407. These components were mixed together with PBS by heating at 50 °C for 15 min with stirring; subsequently, this solution was stirred for 45 min at 45 °C. PTX (1.25 mg) was dissolved in ethanolic solution for loading on zein nanoparticles and the PTX-loaded zein nanoparticle solution was evaporated to remove the organic solvent using a rotary evaporator. Thin film was hydrolyzed with PBS and stirred for 12 h until PTX-loaded non-targeted nanoparticles (PTX/Zein NPs) were obtained. DDC/NHS-based carbodiimide principle was applied for the conjugation of PTX/Zein NPs and FA-PEG-COOH. Briefly, DCC (15 mg), NHS (25 mg), and TEA (10 mg) were added to PTX/Zein NPs to activate the amines in the zein in PTX/Zein NPs. Finally, FA–PEG–COOH (0.5 g) was dissolved in DMSO and added to the activated PTX/Zein NPs. This reaction mix was incubated for 12 h at room temperature in a nitrogenous atmosphere, dialyzed with deionized water, and freeze-dried under vacuum to produce a dry powder of PTX/Zein-FA for further in vitro and in vivo experiments [25].

### 2.4. Characterization of PTX/Zein-FA

#### 2.4.1. Particle Size and Zeta Potential

Particle size and zeta potential of PTX/Zein-FA were determined using dynamic light scattering (DLS). These measurements were carried out in triplicate at 25 °C and a scattering angle of 90° to produce a mean value with the aid of Zeta Sizer (Nano ZS90; Malvern Instruments, Malvern, UK) (Nano DTS software, version 6.34) [26].

#### 2.4.2. Morphological Characterization

A transmission electron microscope (H-7600; Hitachi, Tokyo, Japan) was used to characterize the shape and morphology of PTX/Zein-FA [27]. Briefly, the samples were adsorbed on carbon-coated copper grids and dried at room temperature, after staining with 2% phosphotungstic acid, to visualize the nanoparticles.

#### 2.4.3. FTIR Analysis

FTIR analysis was performed using a Thermo Scientific Nicolet Nexus 670 FTIR spectrometer and Smart iTR with a diamond window (Thermo Fisher Scientific, Waltham, MA, USA). The spectra were observed in the range of 550–4000 cm^−1^ to record specific peaks of the freeze-dried samples [28].

#### 2.4.4. X-ray Diffraction Analysis

To characterize the crystalline nature of the nanoparticle formulation, XRD analysis was carried out. The diffraction patterns of freeze-dried powder of PTX/Zein NPs and PTX/Zein-FA were observed using a vertical goniometer and X-ray diffractometer (X’pert PRO MPD diffractometer, Almelo, The Netherlands) and measured under Ni-filtered Cu Kα-radiation (current 30 mA; voltage 40 kV). Scanning was performed over diffraction angles (2θ) from 10° to 60° ranges at a rate of 5°/min [29]. The resulting patterns of PTX/Zein NPs and PTX/Zein-FA were compared with Zein, PTX, and FA–PEG–COOH.

#### 2.4.5. Colloidal Stability

The stabilities of PTX/Zein NPs and PTX/Zein-FA at storage temperatures of 4 and 25 °C were evaluated by recording the changes in mean size, polydispersity index (PDI), zeta potential, and drug content, at designated time points, for 45 days using DLS [24]. Ionic media with NaCl and PBS were used for the dilution of freeze-dried formulations, PTX/Zein NPs, and PTX/Zein-FA, respectively.

#### 2.4.6. Encapsulation Efficiency and Loading Capacity

High-Performance Liquid Chromatography (HPLC Agilent 1200 series, Agilent Technologies, Inc., Santa Clara, CA, USA) was performed to estimate the encapsulation efficiency (EE) and loading capacity (LC) of PTX in PTX/Zein NPs and PTX/Zein-FA [30]. Unbound PTX was separated from PTX/Zein NPs and PTX/Zein-FA by filtering with an Amicon^®^ centrifugal filtering unit (molecular weight cutoff, 10,000 Da; EMD Millipore, Billerica, MA, USA). Mobile phase containing distilled water and acetonitrile (40:60 v/v) was used at a flow rate of 1.0 mL/min, a C_18_ column (Inertsil^®^ ODS3: 0.5 μm, 15 cm × 0.46 cm, GL Sciences Inc., Tokyo, Japan) was used as the stationary phase, and the resolved components were detected at 227 nm using a UV-Vis detector. The percentage amounts of EE and LC were calculated using following equations.

$$EE\;\left(\%\right)=\frac{W_{D}}{W_{T}}\times 100\;;$$

where, *W*_D_ is the weight of PTX encapsulated in PTX/Zein NPs and PTX/Zein-FA, and W_T_ denotes the total weight of PTX in the formulation.

$$LC\left(\%\right)=\frac{W_{\mathrm{TD}}-W_{\mathrm{UD}}}{W_{\mathrm{TN}}}\times 100\;;$$

where, total weight of PTX; unbound PTX; and total weight of zein nanoparticles are represented as *W*_TD_, *W*_UD_, and *W*_TN_, respectively.

### 2.5. In Vitro Release Study

Dialysis was performed to determine the rate of release of PTX from PTX/Zein-FA. Briefly, dialysis membrane bags (Spectra/Por 3500 Da-MWCO, New Brunswick, NJ, USA) containing 1 mL of formulations each were suspended in 30 mL of different buffer solutions with strict pH range, such as pH 5.0 of acetate-buffered saline (ABS, 0.01 M) and pH 6.5 and pH 7.4 of phosphate-buffered saline (PBS, 0.01 M). At the stated experimental conditions (100 rpm speed and at 37 °C), the samples were collected at specified time points and replaced with fresh buffer media [31].

### 2.6. In Vitro Cytotoxicity

The cytotoxicity of PTX/Zein-FA was estimated in the folate receptor-expressing KB cells and folate receptor-deficient A549 cells by using MTT assay [32]. Both cell lines were seeded in 96-well plates (1 × 10^4^ cells/well) before treatment with free PTX, zein NPs, PTX/Zein NPs, or PTX/Zein-FA and incubated for 24 h at 37 °C in a 5% CO_2_ incubator. After 24 h, MTT was added to each well and the plates were incubated for 4 h; finally, absorbance was measured at 570 nm using an automated microplate reader. The percentage of cell viability was used to determine the efficacy, and cytotoxicity was calculated using the following equation, where untreated cells were used as the control [33].

$$\mathrm{Cell}\;\mathrm{viability}\;(\%)\;=\;\frac{A570\left(sample\right)\;-\;A570\left(blank\right)}{A570\;\left(control\right)\;-\;A570\left(blank\right)}\times \;100;$$

where OD is optical density.

### 2.7. In Vitro Cellular Study

#### 2.7.1. Intracellular Uptake Efficacy

Cellular uptake of PTX/Zein-FA by receptor-mediated endocytosis of PTX/Zein-FA in KB cells was examined by using fluorescence-activated cell sorting (FACS) (BD Biosciences, San Jose, CA, USA) [34], and the results were compared to the folate receptor-deficient A549 cells. Both cell lines were incubated for 24 h after seeding in 6-well plates at a density of 2 × 10^6^ cells/well. Coumarin-6 was used as a fluorescence dye. Free FA (20 µg) was added to one group of each cell line as a pretreatment before the cells were treated with PTX/Zein-FA, to examine the efficacy of folate receptor-mediated uptake of PTX/Zein-FA. Finally, the cells were harvested by trypsinization, washed with PBS, and resuspended in PBS for flow cytometry analysis.

#### 2.7.2. Live Cell Analysis

KB and A549 cells, seeded in 12-well plates at a density of 1 × 10^5^ cells/well, were treated with free PTX, PTX/Zein NPs, or PTX/Zein-FA. After 24 h, PBS was used to wash the cells; subsequently, the cells were stained with fluorescence indicators to evaluate live and dead cells, i.e., propidium iodide and acridine orange, respectively, for 1 h. After staining, cells were washed with PBS and examined using a fluorescence microscope (Nikon Eclipse Ti, Nikon Instruments Inc., Tokyo, Japan) [35].

#### 2.7.3. Cell Cycle Analysis

To analyze the distribution of PTX/Zein-FA in cell cycle phases, both KB and A549 cells were incubated in 12-well plates for 24 h. After 24 h, cells were treated with free PTX, PTX/Zein NPs, or PTX/Zein-FA and incubated for an additional 24 h. Cell-clock reagent was added to each well after treated compounds had been washed with PBS to analyze color changes to dark blue, green, and yellow corresponding to the G2/M, S, and G0/G1 phases. The images were captured using fluorescence microscope (Nikon Eclipse Ti), and the number of positive cells in the individual plane was determined by using ImageJ software 1.52q.

#### 2.7.4. Cell Migration Studies

The wound healing properties of PTX/Zein-FA were investigated in KB and A549 cell lines. Monolayers of cells were scratched with sterile pipette tip to induce a wound after the cells had been incubated in 12-well plates at a density of 2 × 10^5^ cells/well. After incubation for 24 h, cells were washed with PBS and treated with free PTX, PTX/Zein NPs, or PTX/Zein-FA, and untreated cells were used as controls. The migration rate, indicative of wound healing, was observed by measuring the distance between the edges of scraps; and the results for folate receptor-positive cancer cells were compared with that of folate receptor-negative cancer cells [36].

#### 2.7.5. Western Blot Analysis

The expression of proapoptotic and anti-apoptotic proteins in both KB and A549 cells was examined by Western blot analysis. Briefly, the cells were collected to quantify the amount of proteins using BCA Protein Assay Kit (Thermo Fisher Scientific, Waltham, MA, USA) after treatment with free PTX, PTX/Zein NPs, or PTX/Zein-FA. The extracted proteins were electrophoresed on Bis-Tris polyacrylamide gel (10%) and were transferred on polyvinyl fluoride (PVDF) membrane. The membranes were then blocked with the suspension of nonfat milk powder suspension (5%) in TBST followed by overnight incubation with primary antibodies (GAPDH, Bcl2, Bax, pP53, p27, or Caspase 3). Finally, bands of different proteins were observed by using luminol solution (Thermo Fisher Scientific, Waltham, MA, USA) and imaged using enhanced chemiluminescence after incubation with suitable secondary antibodies for 1 h [37].

### 2.8. In Vivo Imaging and Biodistribution Analysis

Three groups of KB tumor-bearing mice were developed with the tumor volumes of 100–200 mm^3^ observed in mice from each group. Free cyanine 5.5, cyanine 5.5-loaded zein nanoparticles with or without folate-targeting ligand (100 μL of each containing 1 μg/mL of Cyanine 5.5) were injected into the mice via the tail vein. At the specified time points, the distribution of injected formulations with fluorescence signals was observed by using the FOBI fluorescence imaging system (Neoscience Co. Ltd., Seoul, Korea). After 24 h, the main organs of the mice (heart, liver, spleen, lungs, kidneys, and tumor) were extracted from the scarified mice and semi-quantitative biodistribution of the fluorescence was analyzed and recorded by ex vivo imaging [38].

### 2.9. In Vivo Antitumor Efficacy

All the experiments in the animal models were performed with the approval of Institutional Animal Ethics Committee Yeungnam University, South Korea (Approval No. 2017-012, 7 August 2017). BALB/c nude mice (females, 6-weeks) were injected subcutaneously in the flank region with KB tumor cells to establish the tumor-bearing xenograft model. The mice were randomly distributed into four groups based on body weight (five mice/group) when the tumor volume reached ~150 mm^3^; they were treated with free PTX, PTX/Zein NPs, or PTX/Zein-FA (8 mg/kg each) intravenously via tail vein injection twice a day for 3 weeks. Tumor volumes were calculated according to the following equation:

$$V\;=\frac{\;(\mathrm{Width}{)}^{2}\;\times \;\mathrm{Length}}{2}$$

where, width and length of tumor were measured with Vernier calipers and the body weights of all the groups were checked daily to determine the toxicity of each treatment. At the termination time points, mice were sacrificed using CO_2_ inhalation and the tumors and main organs of all mice from each group were harvested and fixed in formalin for immunohistochemistry analysis [39].

### 2.10. Histopathological Characterization

KB cells containing tumors were extracted from the mice and were sliced to yield 3–4 µm-thick sections that were used for hematoxylin and eosin staining (H&E) for the characterization of histopathological lesions under the optical microscope (Nikon Corporation, Tokyo, Japan). Furthermore, computer-based automated image analyzer (iSolution FL ver 9.1; IMT i-Solution Inc.; Vancouver, Quebec, Canada) calculated the tumor cell volumes and intact tumor cell-occupied regions (% mm^−2^ of tumor mass) [40]. Specific antibodies with avidin–biotin–peroxidase complex (ABC) and peroxidase substrate kits (Vector Labs, Burlingame, CA, USA) were used to analyze the proliferation index by analyzing the levels of CD31, Ki-67, PARP, (poly (ADP-ribose) polymerase), and caspase-3 in extracted tumors. Tissue sections were incubated for 30 min in methanol and 0.3% H_2_O_2_ to block the activity of nonspecifically-binding immunoglobulins and endogenous peroxidase, and incubated for 1 h in a humidified chamber after inducing heat epitope retrieval (95–100 °C) in 10 mM citrate buffer (pH 6.0) with normal horse serum-blocking solution. These incubated tissue sections were treated with primary antisera overnight at 4 °C and incubated with ABC reagents and biotinylated universal secondary antibodies for 1 h at room temperature. At each successive step, all the samples were washed three times with 0.01 M PBS. An automated image analyzer measured the area (% mm^−2^ of tumor mass) occupied by caspase-3 and PARP-positive cells located in the tumor mass [41].

### 2.11. Statistical Analyses

For determination of statistically significant differences between pairs of groups and multiple groups, Student’s t-tests and one-way analysis of variance (ANOVA) were used. All the results are represented as mean ± standard deviation (SD). *p* < 0.05 was considered statistically significant.

## 3. Results and Discussion

### 3.1. Preparation and Characterization of PTX/Zein-FAs

**Figure 2:**
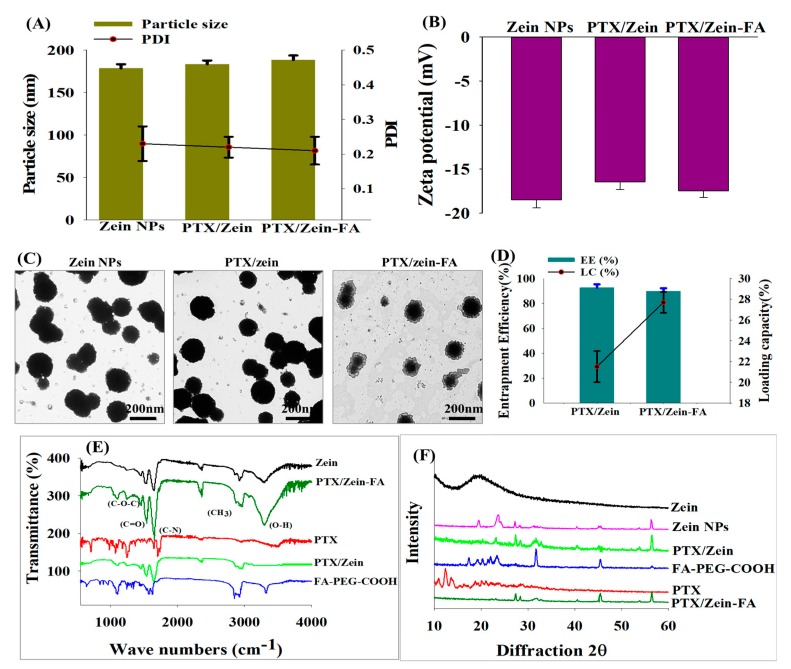
Physicochemical characterization of zein, PTX/Zein, and PTX/Zein-FA NPs. (**A**) Particle size and polydispersity index; (**B**) zeta potential; and (**C**) TEM images of zein NPs, PTX/Zein NPs, and PTX/Zein-FA. (**D**) Percentage entrapment efficiency and loading capacity of paclitaxel in PTX/Zein NPs and PTX/Zein-FA NPs. (**E**) FTIR spectra and (**F**) XRD analysis of different NP formulations.

PTX/Zein-FA nanoparticles were prepared using the solvent evaporation method. After loading PTX on Zein NPs, particle size did not increase significantly (Figure 2A), which supported the speculation that PTX was successfully loaded in nanoparticles and expanded the hydrophobic corona of nanoparticles [42,43]. However, conjugation of FA to PTX/Zein NPs led to a marginal increase in particle size up to 189.0 ± 2.5 nm. All the characterized formulations had negative zeta potential (Figure 2B). The morphology of all nanoparticle formulations had spherical appearance and rough surface; moreover, after conjugation with FA–PEG–COOH, the outer layers were observed in TEM images of PTX/Zein-FA (Figure 2C). Therefore, FA–PEG–COOH was successfully conjugated onto the PTX/Zein NPs [44]. Colloidal stability of PTX/Zein-FA was investigated by observing the particle size, zeta potential, and PDI of freeze-dried form of PTX/Zein-FA under the storage temperatures of 4 and 25 °C over a period of 45 days. DLS analysis showed that PTX/Zein-FA had excellent physical stability (Figure S2), due to the stabilizing effect of surfactants and negative charge of nanoparticles [45,46]. LC and EE of the nanoparticles were determined in both targeted and non-targeted nanoparticles (Figure 2D). EE of PTX/Zein NPs and PTX/Zein-FA had more than 90%, and LC of both nanoparticles were recorded as 21.5 ± 1.5% and 27.7 ± 1.0%, respectively. In order to characterize the chemical functionalization of the PTX/Zein-FA, the FTIR spectrum was compared with that of the individual ingredients used in preparation of PTX/Zein-FA. Figure 2E represents the characteristic peaks of related compounds; in the spectrum of PTX/Zein-FAs, the absorption peaks of (C–O–C), (C=O), and (C=N) were observed at 1290 cm^−1^, 1640 cm^−1^, and 1760 cm^−1^; and at 3430 cm^−1^ the peak represented as an O–H stretching band and the peak related to the asymmetric methyl group (CH_3_) were recorded at 2943 cm^−1^ [47]. XRD pattern of PTX/Zein-FA (Figure 2F) indicated the amorphous nature of obtained nanoparticles when they were compared with the spectra of free PTX due to the possible interaction of hydrogen bonding between FA–PEG–COOH and PTX-loaded zein nanoparticles [48].

**Figure 3:**
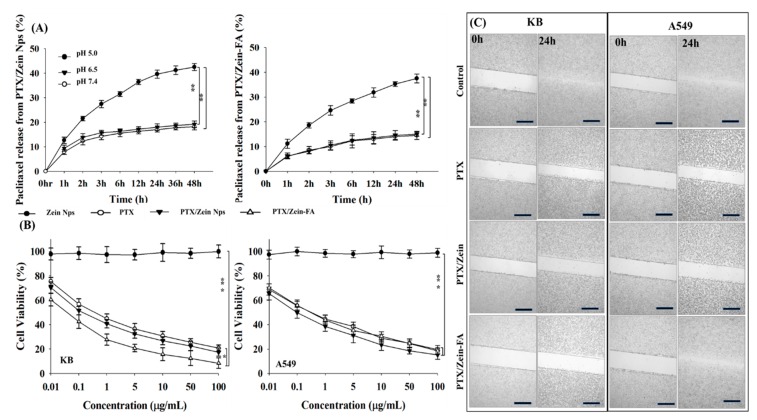
(**A**) In vitro release profiles of PTX from PTX/Zein NPs and PTX/Zein-FA at pH 5.0, 6.5, and 7.4, respectively. (**B**) In vitro cytotoxic effect of free drug and PTX, PTX/Zein, and PTX/Zein-FA NPs at different concentrations on folate receptor-expressing KB cells and folate receptor-deficient A549 cells. Data are shown as the mean ± SD (*n* = 6) (* *p* < 0.05, ** *p* < 0.01, *** *p* < 0.001). (**C**) The effect of PTX, PTX/Zein, and PTX/Zein-FA NPs on wound healing, migration of KB and A549 cells was observed at 0 h and 24 h. Scale bar, 100 µm.

Furthermore, the release kinetic profile of PTX from PTX/Zein-FA was observed at different pH values, PBS (pH 7.4 and 6.5) and ABS (pH 5.0), and the results were compared with the release rate of PTX from PTX/Zein NPs. The release of PTX from both targeted and non-targeted nanoparticles in acidic pH condition showed more increase than that of the physiological pH of 6.5 and 7.4 (Figure 3A). The statistical difference between the release profiles at different pH values due to the swelling capacity of zein matrix in acidic pH supported the hypothesis of this research design to deliver cargo drug in a tumor environment [49]. Moreover, sustained release of PTX from PTX/Zein-FA was confirmed when it was compared with the results from non-targeted nanoparticles [50]. This was possible because of free PTX which showed faster release from non-targeted nanoparticles, though the hydrated swollen matrix of zein and folate-targeted PEG can protect the bioactive molecule inside the nanoparticle from degradation before reaching the targeted area [51].

### 3.2. In Vitro Cellular Assays for PTX/Zein-FA

The cytotoxicity of PTX/Zein-FA was observed in folate receptor-expressing KB cells and folate receptor-deficient A549 cells by MTT assay, and the results were compared with cells treated with free PTX or PTX/Zein nanoparticles (Figure 3B). As expected, the cell viability of folate receptor-expressing KB cells treated with PTX/Zein-FAs was the lowest and the IC_50_ value was also observed to be lower than cells treated with free PTX and PTX/Zein NPs (Table S1). This was because higher uptake of nanoparticles with targeting effect increased the delivery of cargo chemotherapeutic agents. However, in the case of folate receptor-deficient A549 cells, the cytotoxic effect of PTX/Zein-FA was almost equivalent to free PTX, and IC_50_ values were also similar to that of free drug suggesting that the cytotoxicity of the engineered nanoparticles depends on receptor-mediated endocytosis [52].

One of the mechanisms regulating the invasiveness of tumor cells and pathological processes involves their migratory properties. Therefore, we analyzed the migratory patterns of PTX/Zein-FA-treated KB and A549 cells. Migration of KB cells was significantly inhibited after treating with PTX/Zein-FA after 24 h (Figure 3C). However, the cells were noted to be growing in an area between the scratched edges of A549 cells after treatment with PTX/Zein-FA. The inhibition of invasive and adhesive cellular properties supports the wound-healing properties of PTX/Zein-FA, as these nanoparticles were taken up by the receptors on the cells and internalized by endocytosis.

**Figure 4:**
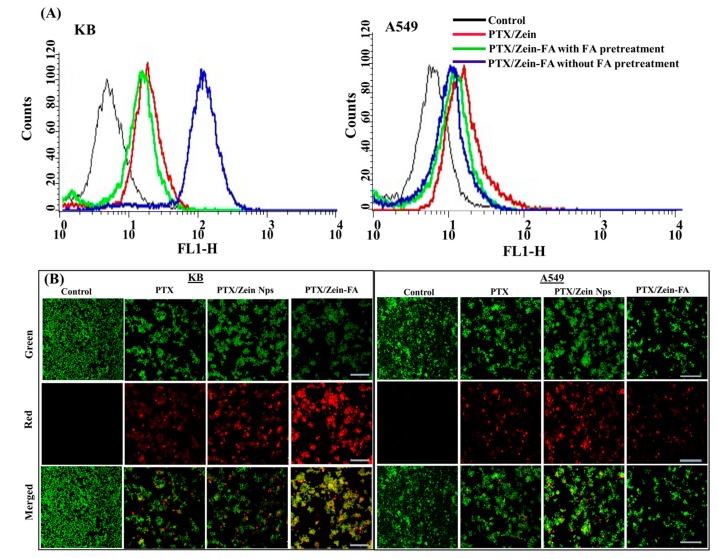
(**A**) Quantitative cellular uptake of coumarin-6-loaded targeted nanoparticles in KB and A549 cells as determined by FACS compared to pretreatment with or without FA. (**B**) Fluorescence imaging of live/dead folate receptor-expressing KB and folate receptor-deficient A549 cells incubated for 24 h with free drug and PTX, PTX/Zein, and PTX/Zein-FA NPs, respectively. Green and red fluorescence represent live and dead cells, respectively. Scale bars, 100 µm.

Cellular uptake efficiency of PTX/Zein-FA in both the cell lines was examined by flow cytometry analysis (Figure 4A). The uptake of PTX/Zein-FA in folate receptor-expressing KB cells was observed to increase with increasing time and dose of treatment, whereas the levels of uptake in folate receptor-deficient A549 cells did not show significant differences with regards to time and dose, respectively (Figure S3). Therefore, receptor-mediated endocytosis of PTX/Zein-FA supports the cellular uptake efficiency of PTX/Zein-FA in both the cell lines. Furthermore, free FA pretreatment significantly reduced cellular uptake of PTX/Zein-FA, which confirmed that cellular uptake was predominantly via receptor-mediated endocytosis. The imaging for live and dead cells are shown in (Figure 4B) after staining with acridine orange (AO; indicator of live cells) and propidium iodide (PI; staining for dead cells). The live/dead assay shows that the intensity of red fluorescence in the PTX/Zein-FA-treated group was higher than any other treatment groups, which indicated that PTX/Zein-FA nanoparticles showed superior effect as compared to free PTX and non-targeted PTX/Zein nanoparticles in folate receptor-expressing KB cells. However, red fluorescence in folate receptor-deficient A549 cells after treatment with PTX/Zein-FA did not show an increase, indicating that immobilization of folic acid in PTX/Zein-FA does not have a higher cytotoxic effect on folate receptor-deficient cell lines [53].

**Figure 5:**
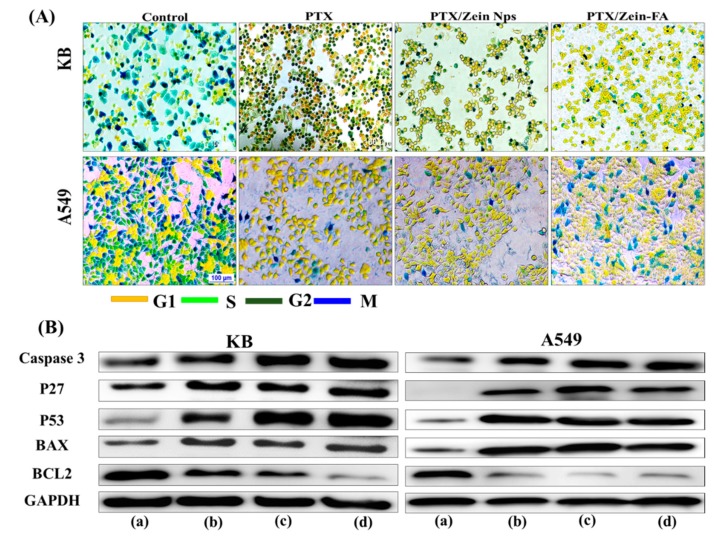
(**A**) Fluorescence microscopic analysis of cell phase distribution of free drug and PTX, PTX/Zein, and PTX/Zein-FA NPs on folate receptor-positive KB and folate receptor-deficient A549 cells after 24 h of incubation. Scale bars, 100 µm. (**B**) Western blot analysis of KB and A549 cells following incubation with free drug and PTX, PTX/Zein, and PTX/Zein-FA NPs using the indicated protein markers. GAPDH was used as loading control.

The effect of PTX/Zein-FA in cancer cells was characterized by studying the distribution of PTX/Zein-FA in major cell cycle phases of both cell lines, KB and A549 (Figure 5A and Figure S4A). The cell cycle data indicates that PTX is a potent inhibitor of cell development in G2 and/or M phase of cell cycle in both cell lines, KB and A549. In folate receptor-expressing KB cells, the number of cells arrested in G2 and/or M phase was higher than that of free PTX and non-targeted NP-treated cells, but in the case of folate receptor-deficient A549 cells, the percentage of cells in G2 and/or M phase was similar to that of drug-free and untargeted nanoparticles. The possible explanation for maximum arrest of KB cells in G2 and/or M phase upon treatment with PTX/Zein-FA could be attributed to apoptosis.

Apoptotic, antiapoptotic, and cell cycle-related protein levels were examined in PTX/Zein-FA-treated KB cells (Figure 5B and Figure S4B). The results obtained were confirmed by comparing PTX/Zein-FA-treated KB cells with free PTX or non-targeted NPs treated cells. Moreover, A549 cells were used to prove that the targeting effect of nanoparticles promoted apoptosis and microtubule stabilization due to the presence of PTX in the formulation. It has been demonstrated that PTX/Zein-FA increased the levels of cell cycle proteins, p53 and p27, in KB cells leading to cells arresting in G2 and/or M phases of the cell cycle. Furthermore, the levels of apoptotic markers caspase-3 and Bax were significantly elevated; and anti-apoptotic protein, Bcl-2, was remarkably decreased in PTX/Zein-FA-treated KB cells. However, the expression levels of these apoptotic and anti-apoptotic proteins were not significantly different as compared to PTX/Zein-FA-treated A549 cells when compared with that of free PTX and non-targeted groups. Therefore, the Western blot data suggests that folate-targeted zein nanoparticles could transport PTX into cells via the folate receptors in cancer cells.

### 3.3. In Vivo Imaging and Biodistribution Study

**Figure 6:**
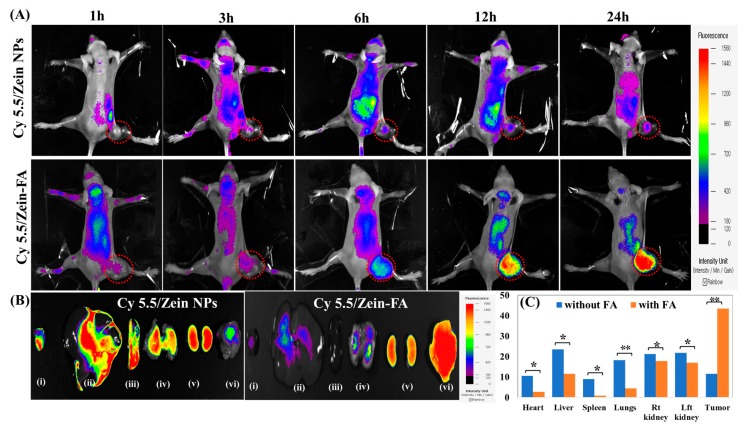
Biodistribution studies in KB tumor-bearing mice. (**A**) In vivo fluorescence imaging of subcutaneous injection of KB cells in BALB/c nude mice after an intravenous injection of Cy5.5-loaded Zein NPs with or without the folate-targeting ligand (both containing 1 μg/mL cyanine 5.5). (**B**) Ex vivo images of dissected organs from tumor-bearing mice sacrificed 24 h after intravenous injection of Cy5.5-loaded zein NPs with and without folate-targeting ligand (both containing 1 μg/mL cyanine 5.5). (**C**) Calculation of fluorescence intensity in various organs (heart, liver, spleen, lungs, and kidneys) and tumors from the sacrificed mice (* *p* < 0.05, ** *p* < 0.01).

Active targeting effect of PTX/Zein-FA in a localized tumor area was analyzed by examining the distribution patterns of nanoparticle formulations in KB tumor-bearing mice. Although fluorescence from both treated groups was immediately distributed throughout the circulation according to time-dependent fluorescence images, the fluorescence signal in the tumor area of the Cy5.5/Zein-FA-treated group was about two times higher than that of Cy5.5/Zein NPs after a treatment of 24 h (Figure 6A), indicating that the accumulation of nanoparticles in the tumor area due to the targeting effect of nanoparticles improved the drug delivery system. After 24 h, mice were sacrificed and tumor tissues and all main organs, such as liver, kidneys, heart, lungs, and spleen were extracted from each treated group to detect the fluorescence intensity in an ex vivo study. Although fluorescence from Cy5.5/Zein-FA showed distribution in liver, lungs, heart, and kidneys due to the circulation of nanoparticles, fluorescence intensities in these organs were significantly lower than that from the Cy5.5/Zein NP-injected group (Figure 6B,C). Therefore, folate-targeted nanoparticles show maximum accumulation in folate receptor-expressing tumors with minimum cardiotoxicity due to enhanced permeability and retention effect and receptor-mediated endocytosis.

**Figure 7:**
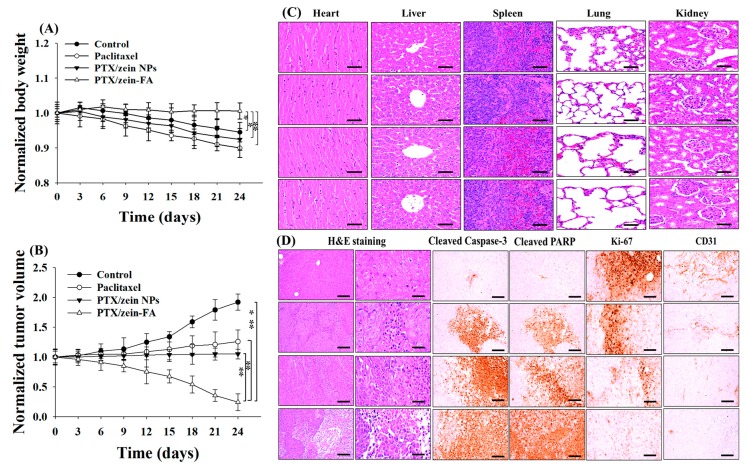
In vivo antitumor studies on (**A**) tumor volume and (**B**) body weight in KB tumor-bearing BALB/c nude mice following intravenous administration of different formulations (control, free drug and PTX, PTX/Zein, and PTX/Zein-FA NPs). Each formulation was administered four times at 3-day intervals (* *p* < 0.05, ** *p* < 0.01). (**C**) Histopathological changes in the heart, liver, spleen, lungs, and kidneys, and (**D**) immunohistopathological analyses. Representative changes in tumor histopathology and immunoreactivity to caspase-3, PARP, Ki-67, and CD31 in tumor mass. Caspase-3 and PARP are markers for apoptosis, and CD31 and Ki-67 are markers for angiogenesis. Scale bars, 120 μm.

To examine the effectiveness of PTX/Zein-FA, KB tumor-bearing xenograft mice were used for in vivo antitumor study. We observed that folate-targeted NPs significantly promoted the antitumor efficacy of PTX in folate receptor overexpressing cancer cells, because the rate of tumor growth and volume of tumor cells were significantly inhibited in PTX/Zein-FA-treated group when compared to free PTX and non-targeted NP treatment groups (Figure 7A). The body weight of mice from the PTX/Zein-FA treatment group remained almost constant until the end of the study period, which shows almost no toxicity at this dose (Figure 7B). This was confirmed by H&E staining of principal organs with no toxicological effects at the histopathological level (Figure 7C, Table S2). However, body weights of mice from free PTX and PTX/Zein NPs without targeting nanoparticles-treated groups showed a remarkable decrease, which could be due to the higher distribution of free PTX to normal tissues. Moreover, H&E staining for histopathological analysis showed that PTX/Zein-FA treatment inhibited mechanisms of tumor cell proliferation and angiogenesis (Figure 7D, Table S3). PTX/Zein-FA-treated groups had low levels of angiogenesis and cell proliferation markers, Ki-67 and CD31, whereas the levels of apoptotic markers PARP and caspase-3, showed significant increase as compared to the results obtained from groups treated with free PTX and non-targeting nanoparticles. Promotion of tumor cell apoptosis and abatement of angiogenesis and proliferation were mainly due to the effect of folate-targeting PEG which caused an accumulation of PTX in the targeted tumor area and caused DNA fragmentation accompanied with apoptosis to inhibit tumor progression. In vivo antitumor study confirmed that the engineered PTX/Zein-FA showed maximum antitumor efficacy by improving cellular apoptosis and retarding tumor cell proliferation via active targeting mechanism and high accumulation of chemotherapeutic agent, PTX, in the targeted tumor area via folate-targeted zein nanoparticles.

## 4. Conclusions

PTX-loaded folate-targeted zein nanoparticles were successfully prepared with nanosized spherical morphology. Folate-targeted zein nanocarriers could deliver PTX to specific tumor cells via folate receptor-mediated endocytosis and could extend into systemic circulation with the help of PEG in a folate-conjugated ligand based on the results obtained in in vitro and in vivo experiments. The potential advantages of drug carrier, zein and targeting effect of folate-targeted PEG include high cellular uptake of the drug, induction of apoptosis, and inhibition of cell migration in folate receptor-expressing KB cells compared to folate receptor-deficient A549 cells, according to the in vitro experiments. The specific antitumor activity of the PTX/Zein-FA was confirmed in folate receptor-overexpressing KB tumor-bearing mice in the in vivo study. Altogether, it demonstrated that PTX/Zein-FA could be potent therapeutic nanocarriers for safe and effective chemotherapy.

## Supplementary Materials

The following are available online at https://www.mdpi.com/1999-4923/11/11/562/s1, Figure S1: Synthesis and characterization of folate-PEG. (**A**) Synthesis scheme of FA-PEG-COOH. (**B**) FTIR spectra of NH_2_-PEG-COOH, folic acid, and FA-PEG-COOH. (**C**) ^1^H-NMR spectra of FA-PEG-COOH, Figure S2: Stability tests of PTX/Zein NPs and PTX/Zein-FA for 45 days at two different temperatures of storage conditions by measuring particle sizes, Figure S3: Comparison of time-dependent and concentration-dependent cellular uptake efficiency of PTX/Zein-FA in KB and A549, Figure S4: Quantitative evaluation of (**A**) cell cycle distribution of free drug, PTX, PTX/Zein NPs, and PTX/Zein-FA, and (**B**) levels of different protein markers in folate receptor-expressing, KB and folate receptor-deficient A549 cell lines, Table S1: IC 50 value of Paclitaxel and final formulation, PTX/Zein-FA in KB and A549 cell lines, Table S2: Histopathological-histomorphometrical Analysis of Principal Organs, Taken from KB Tumor Cell Xenograft Athymic Nude Mice. Table S3: Histomorphometrical analysis of tumor masses, taken from KB tumor cell xenograft athymic nude mice.

## Abbreviation

ABS	Acetate-buffered saline
AO	Acridine orange
DL	Drug loading
DLS	Dynamic light scattering
DMSO	dimethyl sulfoxide
DCC	*N*’,*N*’-dicyclohexyl carbodiimide
EE	Encapsulation efficiency
EDC	1-ethyl-3-(3-dimethyl amino propyl) carbodiimide hydrochloride
FACS	Fluorescence-activated cell sorting
FDA	Food and Drug Administration
FTIR	Fourier-transform infrared spectroscopy
HPLC	High performance liquid chromatography
H&E	Hematoxylin and eosin staining
^1^H NMR	proton nuclear magnetic resonance
LC	loading capacity
MTT	3-(4,5-dimethylthiazol-2-yl)-2,5-diphenyltetrazolium bromide
NHS	*N*-hydroxy succinimide
PTX	Paclitaxel
PTX/Zein NPs	Paclitaxel loaded zein nanoparticles
PTX/Zein-FA	Paclitaxel loaded zein nanoparticles conjugated with folate
PBS	Phosphate-buffered saline
PEG	Polyethylene Glycol
PI	Propidium iodide
XRD	X-ray diffraction
