# Supplementary material for: Development of Folate-Functionalized PEGylated Zein Nanoparticles for Ligand-Directed Delivery of Paclitaxel
Source: Pharmaceutics. 2019 Oct 30;11(11):562. doi: 10.3390/pharmaceutics11110562 (PMC6920870; doi:10.3390/pharmaceutics11110562)
Supplement: Supplementary file 1 [file pharmaceutics-11-00562-s001.pdf]

pharmaceutics

Supplementary Materials: Development of Folate-Functionalized PEGylated Zein Nanoparticles for Ligand-Directed Delivery of Paclitaxel

Zar Chi Soe, Wenquan Ou, Milan Gautam, Kishwor Poudel, Bo Kyun Kim, Le Minh Pham, Cao Dai Phung, Jee-Heon Jeong, Sung Giu Jin, Han-Gon Choi, Sae Kwang Ku, Chul Soon Yong and Jong Oh Kim

1. Synthesis and Characterization of Folate-PEG

FA–PEG–COOH was synthesized by direct conjugation of carboxylic group of folic acid to the amine group of NH_2–PEG–COOH, as illustrated in Figure S1A. FTIR and ^{1}HNMR characterized the conjugated FA–PEG–COOH (Figure S1B) revealed that the FTIR spectrum of FA, NH_2–PEG–COOH and FA-PEG-COOH where specified absorption peaks related to FA and NH_2–PEG–COOH such as -C=O bond occurred at 1647.95 cm^{-1}, C–O–C stretch was at 1448.66 cm^{-1} and the peak of CH_3 was noticed at 3420 cm^{-1}.

Moreover, covalent linkage between FA and NH_2–PEG–COOH was confirmed by H-NMR spectrum (Figure S1C). According to the ^{1}HNMR spectrum of FA–PEG–COOH, the characteristic peaks of the protons of PEG chain, -O-CH_2-CH_2, OH and aminated FA were recognized at δ = 7.49, 7.64, 6.64, 4.45 and 3.31, ppm respectively; and at δ = 8.68, $CH_2CONHCH_2$, new amide linkage of FA and NH_2-PEG–COOH conjugation was observed. Therefore, FA was definitely conjugated with NH_2–PEG–COOH in the synthesis of the folate-targeted amine-PEG-carboxylic acid. Furthermore, the amount of FA in NH_2–PEG–COOH was estimated by using the UV visible spectrophotometry method, and the wavelength used in this measurement was 365 nm. From the result, 39.65% of NH_2–PEG–COOH and 60.35% of FA–PEG–COOH were mixed together in our synthesis of folate-targeted amine-PEG-carboxylic acid. From all these results, we can confirm that FA–PEG–COOH was successfully synthesized.

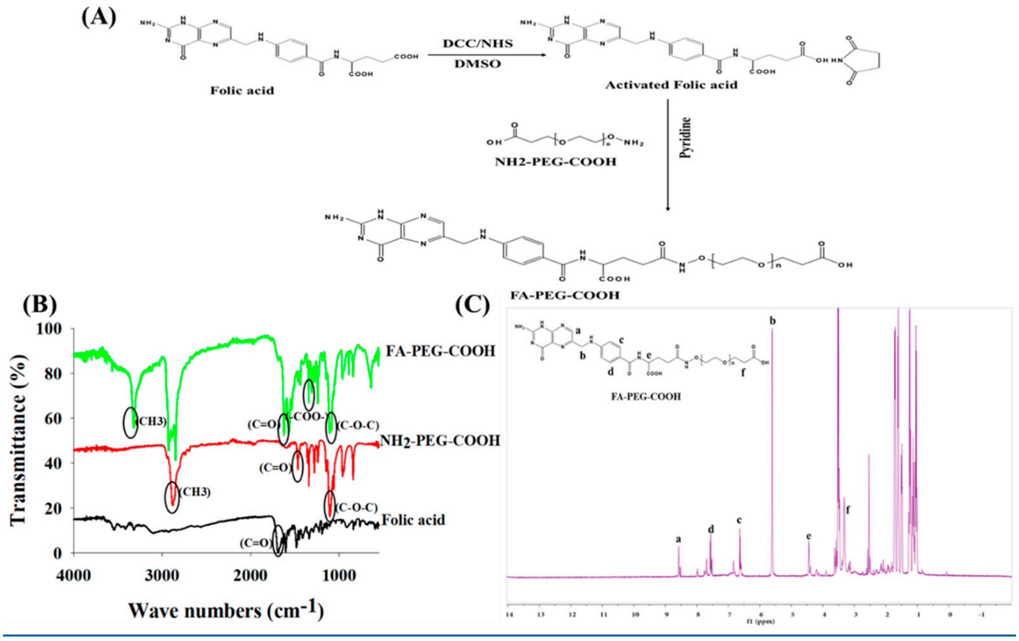

Figure S1. Synthesis and characterization of folate-PEG. (**A**) Synthesis scheme of FA-PEG-COOH. (**B**) FTIR spectra of NH_2-PEG-COOH, folic acid, and FA-PEG-COOH. (**C**) ^{1}H-NMR spectra of FA-PEG-COOH. The results indicate the successful synthesis of folate-conjugated bifunctional PEG as FA-PEG-COOH.

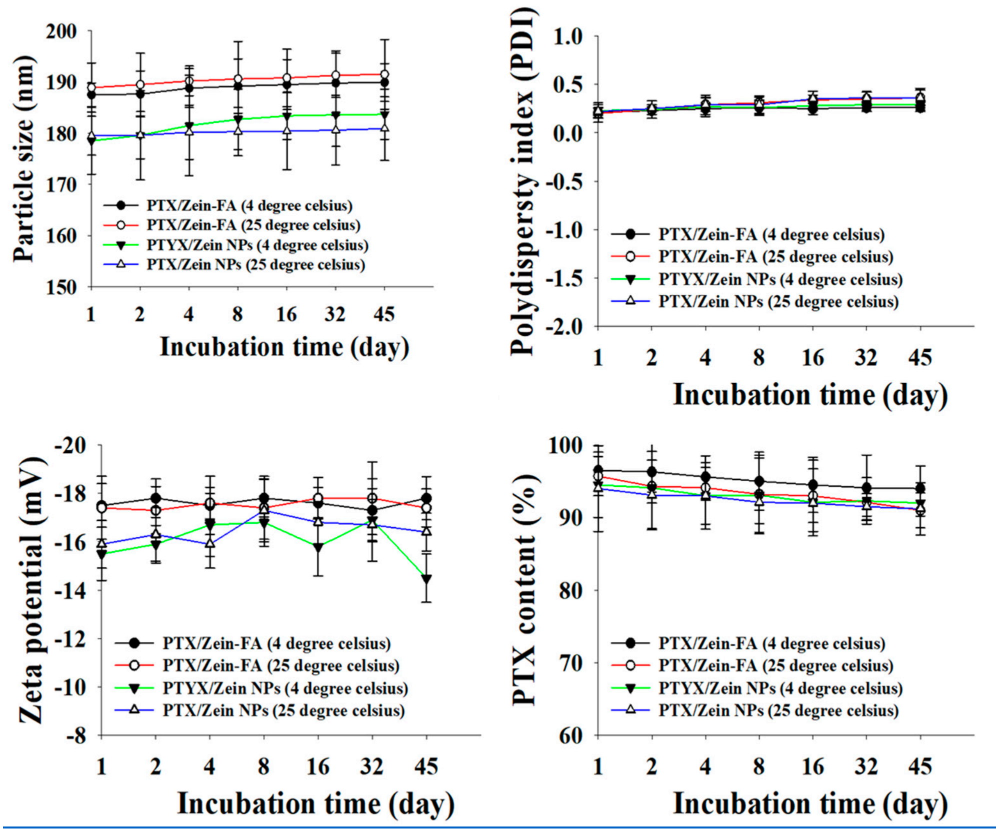

Figure S2. Stability tests of PTX/Zein NPs and PTX/Zein-FA for 45 days at two different temperatures of storage conditions by measuring particle sizes, PDI, zeta potential, and drug contents at 4 and 25 °C.

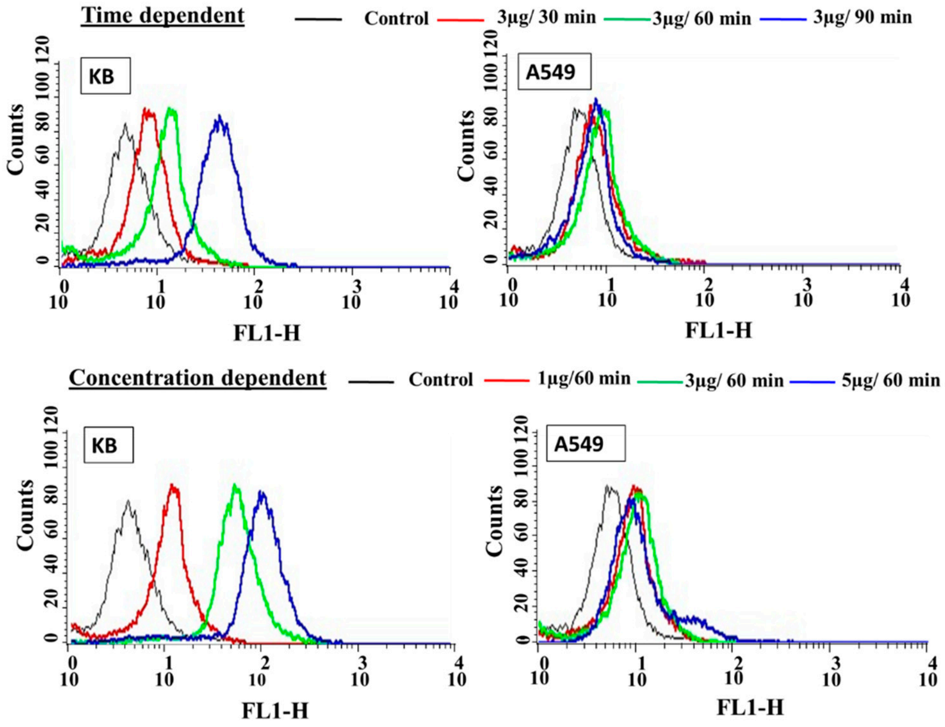

Figure S3. Comparison of time-dependent and concentration-dependent cellular uptake efficiency of PTX/Zein-FA in KB and A549.

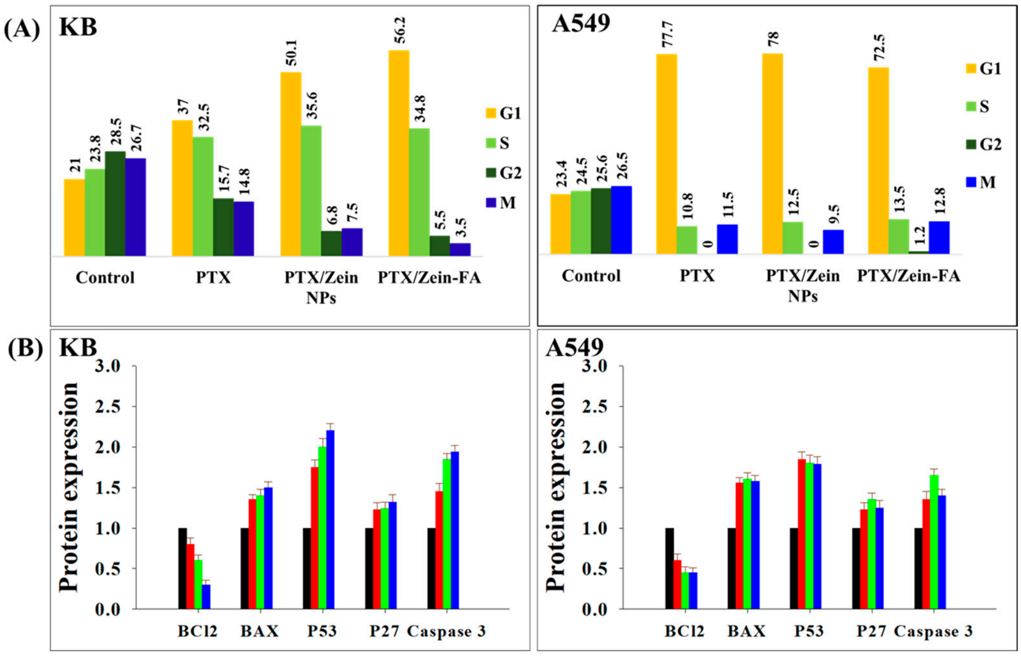

Figure S4. Quantitative evaluation of (**A**) cell cycle distribution of free drug, PTX, PTX/Zein NPs, and PTX/Zein-FA, and (**B**) levels of different protein markers in folate receptor-expressing, KB and folate receptor-deficient A549 cell lines.

Table S1. IC 50 value of Paclitaxel and final formulation, PTX/Zein-FA in KB and A549 cell lines.

Cell lines	IC50 (µg/mL)	
	Paclitaxel (PTX)	PTX/Zein-FA
KB	0.58± 0.20	0.04 ± 0.01
A549	0.39 ± 0.13	0.38 ± 0.01

Table S2. Histopathological-histomorphometrical Analysis of Principal Organs, Taken from KB Tumor Cell Xenograft Athymic Nude Mice.

Organs Groups	Heart Abnormal Finding	Liver Abnormal Finding	Spleen Abnormal Finding	Lung Abnormal Finding	Kidney Abnormal Finding
Control (G1)	0/6 (0%)	0/6 (0%)	0/6 (0%)	0/6 (0%)	0/6 (0%)
Treatment					
G2	0/6 (0%)	0/6 (0%)	0/6 (0%)	0/6 (0%)	0/6 (0%)
G3	0/6 (0%)	0/6 (0%)	0/6 (0%)	0/6 (0%)	0/6 (0%)
G4	0/6 (0%)	0/6 (0%)	0/6 (0%)	0/6 (0%)	0/6 (0%)

Values were numbers of abnormal fields/total observed fields (Six histological fields in each group)

Groups: G1 = KB tumor cell-xenograft vehicle control, five types of principal organs; G2 = Free PTX treated tumor cell-xenograft five types of principal organs; G3 = PTX/Zein NPs treated tumor cell-xenograft five types of principal organs; G4 = PTX/Zein-FA treated tumor cell-xenograft five types of principal organs

SD = Standard deviation; PTX = Paclitaxel; PTX/Zein-FA = PTX/Zein NPs with folate PEG; PTX/Zein NPs = PTX loaded zein nanoparticles

Table S3. Histomorphometrical analysis of tumor masses, taken from KB tumor cell xenograft athymic nude mice.

[Group Summary]

Items<br>Groups	Tumor Cell Volumes (%/mm²)	Immunoreactive Cell Percentages (%/mm² of Tumor Mass)			
		**Cleaved Caspase-3**	**Cleaved PARP**	**Ki-67**	**CD31 (PECAM-1)**
Control(G1)	83.22 ± 10.37	8.90 ± 2.21	5.68 ± 2.83	68.50 ± 10.97	50.22 ± 5.30
Treatment					
G2	62.18 ± 7.12[a]	26.74 ± 7.25 [a]	24.69 ± 7.22 [d]	48.27 ± 6.00 [d]	32.24 ± 5.63 [a]
G3	40.64 ± 5.21[ab]	51.58 ± 7.59 [ab]	48.34 ± 11.09 [de]	36.54 ± 4.95 [de]	17.78 ± 4.18 [ab]
G4	28.01 ± 5.28[abc]	71.71 ± 5.55 [abc]	73.67 ± 7.93 [def]	21.39 ± 2.59 [def]	5.92 ± 2.61 [abc]

Values are expressed as mean ± SD of six tumor mass histological fields

Groups: G1 = KB tumor cell-xenograft vehicle control masses; G2 = Free PTX treated tumor cell-xenograft masses; G3 = PTX/Zein NPs treated tumor cell-xenograft masses; G4 = PTX/Zein-FA treated tumor cell-xenograft masses

PARP = Poly(ADP-ribose) polymerase; CD31 = Platelet endothelial cell adhesion molecule 1 (PECAM-1); SD = Standard deviation; PTX = Paclitaxel; PTX/Zein-FA = PTX/Zein NPs with folate PEG; PTX/Zein NPs = PTX loaded zein nanoparticles

[a] $p < 0.01$ as compared with G1 by LSD test

[b] $p < 0.01$ as compared with G2 by LSD test

[c] $p < 0.01$ as compared with G3 by LSD test

[d] $p < 0.01$ as compared with G1 by MW test

[e] $p < 0.01$ as compared with G2 by MW test

[f] $p < 0.01$ as compared with G3 by MW test

© 2019 by the authors. Submitted for possible open access publication under the terms and conditions of the Creative Commons Attribution (CC BY) license (http://creativecommons.org/licenses/by/4.0/).
